# New Insights into the Research of Bioactive Compounds from Plant Origins with Nutraceutical and Pharmaceutical Potential

**DOI:** 10.3390/plants12020258

**Published:** 2023-01-05

**Authors:** Ivayla Dincheva, Ilian Badjakov, Bistra Galunska

**Affiliations:** 1 Department of Agrobiotechnologies, Agrobioinstitute, Agricultural Academy, 8 Dragan Tsankov blvd., 1164 Sofia, Bulgaria; 2 Department of Biochemistry, Molecular Medicine and Nutrigenomics, Faculties of Pharmacy, Medical University “Prof. Dr. Paraskev Stoyanov”, 84 Tzar Osvoboditel Str., 9000 Varna, Bulgaria

Plant bioactive compounds are essential for human health due to their multiple biological effects, such as antioxidant, anticarcinogenic, antiallergenic, anti-inflammatory, antimutagenic, and antimicrobial activities, which can have beneficial effects on various noncommunicable diseases, such as autoimmune, inflammatory, cardiovascular, cancer, metabolic, and neurodegenerative diseases. Identifying these components and establishing their beneficial health effects are extremely popular activities of scientific inquiry. The screening of natural sources for novel biologically active metabolites has been an essential part of several drug discovery programs.

The aim of this Special Issue is to present the recent developments in high-throughput and efficient analytical approaches, which have enabled the identification of plant-based compounds, the establishment of new protocols for the evaluation of bioactivities, and the development of methods for the extraction, isolation, and structural characterization of new bioactive components with nutraceutical and therapeutic potential.

Three papers in this Special Issue demonstrate the beneficial effects of bioactive compounds from natural sources such as *Pinus* spp., *Ranunculus* spp., and *Galphimia* spp. It has been proved that these components play an important role in the prevention of multiple pathologies. Some of them reduce the risk of numerous diseases, such as, for example, cancer, diabetes, and Alzheimer’s dementia, due to their strong antioxidant activity, whereas others stimulate defense mechanisms, thereby enhancing the response to oxidative stress and preventing widespread damage. Pinosylvin has been shown to perform numerous and diverse actions through its ability to block, interfere, and/or stimulate the major cellular targets responsible for several disorders [1]. Antibacterial and antiprotozoal effects, immunomodulatory and anticarcinogenic properties, and the anti-inflammatory and analgesic actions of *Ranunculus* spp. plants used in traditional medicine applications have been confirmed [2]. *Galphimia* spp. is an important medicinal plant in Mexico, which has many applications in traditional medicine. The main investigations have focused on its anxiolytic and sedative effects due to the presence of the modified triterpenoids, galphimines. However, the genes encoding the enzymes of the galphimine synthesis pathway are still unknown. Hence, in this study, a comparative transcriptome analysis between two contrasting populations of *Galphimia* spp.—a galphimine producer and a non-galphimine producer—is performed using RNA-Seq with the Illumina NextSeq 550 platform to identify putative candidate genes that encode the enzymes of this metabolic pathway. Most of the transcripts were grouped at the molecular function level of gene ontology. In galphimine-producer plants, a larger number of highly expressed transcripts related to acyclic and polycyclic terpene synthesis were identified. As putative candidate genes involved in the galphimine synthesis pathway, P450 family members and enzymes with kinase activity were identified [3].

Essential oils have been used for centuries by many cultures around the world for various purposes, including perfumery, aromatherapy, cosmetics, medicine, feed, bactericides, insecticides, fungicides, etc. Constant research in these areas represents a search for alternatives for more efficient drugs, as well as a way to obtain new products and supplies. Six papers provide the most recent perspectives on the impacts of essential oils on human health and disease.

One article presents the essential oil yield, composition, and bioactivity of *Sagebrush* spp. from the Bighorn Mountains [4]. There was significant variability in the chemical profile and antioxidant capacity between species and subspecies. However, none of the sagebrush EOs had significant antimicrobial, antimalarial, or antileishmanial activity, nor did they contain podophyllotoxin. The results obtained showed an immense chemical diversity that presents an opportunity for the selection of varieties with high concentrations of EOs with desirable compositions. These chemotypes can be selected and possibly introduced into a culture to be grown for the commercial production of these compounds to meet specific industry needs. Another two manuscripts describe the antioxidant and antimicrobial activities of *Thymus vulgaris* L. [5] and *Syzygium aromaticum* L. [6], which are essential oils produced in Slovakia. An in situ antifungal analysis of food models shows that the vapor phase of these oils can inhibit the growth of the microscopic filamentous fungi of the genus *Penicillium*. Additionally, the evaluation of the changes in the biofilm structure of microorganisms on glass and wood surfaces, such as *Salmonella enteritidis, Pseudomonas fluorescens, Pseudomonas expansum,* and *Pseudomonas chrysogenum*, were performed using MALDI-TOF MS. Due to the properties of the tested *T. vulgaris Syzygium aromaticum* L. essential oils, they can be used in the food industry as natural supplements to extend the shelf life of the foods. One study describes the metabolite profile of *Artemisia annua* L. [7], which was grown wild in Romania for the first time. Subsequently, a simple and inexpensive nanocarrier system was developed that capitalizes on the therapeutic properties of both the whole plant and magnetic Fe_3_O_4_ nanoparticles. Further studies are necessary to investigate the biological properties and the bioavailability of the new nanocarrier system. A recent investigation determined the composition of the essential oil and hydrolate of *Dracocephalum moldavica* L. [8], which was grown in the Republic of Serbia and distilled under semi-industrial conditions to determine the in vitro antioxidant and antimicrobial activities. The oil demonstrated better biological activities compared with the hydrolates against *Staphylococcus aureus, Escherichia coli, Salmonella Typhimurium,* and *Listeria monocytogenes,* which represent sources of food-borne illness at the global level. One paper compares the essential oil composition and antimicrobial activity of *Helichrysum arenarium* Moench and *Helichrysum italicum* G. Don, which already have an established international market [9]. Overall, the EO of *H. italicum* from all locations was more effective against *Staphylococcus aureus*. A moderate antimicrobial effect was found against *Candida krusei* and *Candida tropicalis*. The lowest antimicrobial activity was found against *Yersinia enterocolitica*. In general, the tested EOs were more effective against Gram-positive bacteria.

Eight manuscripts covered the topics of analytical strategies for bioactive compound identification and quantification, herbal preparations and natural medicines, and the in vivo and in vitro bioactivity of botanicals. One paper clearly demonstrated the immunostimulation, hematopoietic, and antiviral potential of *Sambucus ebulus* L.’s fruit aqueous extract (FAE), with attention given to its endoplasmic reticulum (ER) stress-reducing potential [10]. J774A.1 macrophages were treated with SE FAE alone or under the condition of lipopolysaccharide (LPS) stimulation. The phytochemical composition of the herbal extracts was analyzed by using GC-MS and LC–MS/MS. Transcription and protein levels were defined using qPCR and Western blot, respectively. Extracts exerted an immunostimulation potential by stimulating IL-6, TNFα, Ccl2, COX2, and iNOS transcription without inducing ER stress. The SE FAE suppressed the LPS-induced transcription of inflammation-related genes (IL-1β, IL-6, TNFα, Ccl2, Icam-1, Fabp4, COX2, iNOS, Noxo1, IL-1ra, and Sirt-1) and reduced the protein levels of iNOS, peIF2α, ATF6α, and CHOP. The effects were comparable to that of salicylic acid. SE suppresses LPS-stimulated inflammatory markers at the transcription and translation levels. The targeting of ER stress is possibly another mechanism underlying its anti-inflammatory potential. These findings reveal the potential of SE as being a beneficial therapeutic for inflammation and ER-stress-related pathological conditions.

Jacaranones are a small group of specific plant metabolites with promising biological activities. Moreover, they can serve as chemotaxonomic markers. The phytochemical investigation of *Crepis pulchra* L. (Asteraceae) resulted in three jacaranone derivatives (jacaranone; 2,3-dihydro-2-hydroxyjacaranone; and 2,3-dihydro-2-methoxyjacaranone) [11]. This is the first report on the isolation of jacaranones from a species belonging to the Cichorioideae subfamily of Asteraceae. Jacaranone derivatives were subjected to an in vitro antiproliferative assay against a panel of human cancer cell lines (MCF-7, MDA-MB-231, HeLa, and C33A), revealing high or moderate activities. Jacaranone (2) showed the highest antiproliferative activity against MDA-MB-231 (human breast cancer) and C33A (human cervical cancer) cells. The volatile compounds of eight peach varieties grown in Bulgaria (*Prunus persica* L.), including “Filina”, “Gergana”, “Ufo-4”, “July lady”, “Laskava”, “Flat Queen”, “Evmolpiya”, and “Morsiani 90”, were analyzed for the first time [12]. Gas chromatography–mass spectrometry (GC–MS) analysis and the HS-SPME technique revealed the presence of 65 volatile compounds. According to the provided principal component analysis (PCA) and hierarchical cluster analysis (HCA), the relative quantities of the identified volatile components depended on the studied peach variety. The results obtained could be successfully applied to the metabolic chemotaxonomy of peaches. Studies regarding the pharmaceutical potential of derivative products (polysaccharides and polyphenols) from plantain displayed prominent antioxidant, antifungal, antitumor, and prebiotic activities (particularly for the polysaccharide fraction) [13]. A comparative phytochemical investigation of *Aronia melanocarpa* L. fruit juices on the Bulgarian market clearly present with a high product quality due to their health-promoting compounds, such as phenolic acids, proanthocyanins, and anthocyanins, which define them as functional foods [14]. Promising new findings have been reported and discussed through the frame of the anti-inflammation, antiandrogen, and hair-growth-promoting potential of shallot (*Allium ascalonicum* L.) extract [15]. One publication clearly presents the antidiabetic activity of Mediterranean edible plant materials using the DIA-DB inverse virtual screening web server. Authors founded that the flavonoids are the most active phytochemicals as they modulate the function of 17 protein targets and present a high structural similarity to antidiabetic drugs. Their antidiabetic effects are linked with three mechanisms of action, namely: (i) the regulation of insulin secretion/sensitivity, (ii) the regulation of glucose metabolism, and (iii) the regulation of lipid metabolism [16]. Molecular docking studies reveal that the Swingle can be a potential source of natural products due to its antibiotic-potentiating activity and since it is anti-SARS-CoV-2 [17].

In recent decades, bioactive compounds from natural sources have attracted substantial attention and have been subjected to extensive research due to their antioxidant properties and their use potential in the promotion of health and the prevention of disease. Some of the most studied topics, in this regard, represent the focus of this Special Issue.
